# Exploring dynamics in catastrophic health care expenditure in Nigeria

**DOI:** 10.1186/s13561-022-00366-y

**Published:** 2022-03-23

**Authors:** Henry C. Edeh

**Affiliations:** Department of Economics, University of Nigeria, Nsukka, Nigeria

**Keywords:** Catastrophic healthcare expenditure, Out-of-pocket payment, Inequality

## Abstract

**Background:**

The Nigeria’s National Health Insurance Scheme aimed at making health care accessible and affordable since it’s became operational in 2005. However, many Nigerians still pay out of pocket for medical expenses, and this drive them to incurring catastrophic health expenditures. Although monitoring progress towards UHC is crucial, one single study exploring the dynamics in catastrophic health expenditure proportion, associated factors, inequality concentration, inequality size, together with decomposition using a longer period Nigeria panel household survey datasets is very scarce.

**Methods:**

Data was drawn from three rounds of the Nigeria General Household Survey. The fixed percentage and rank-dependent thresholds were used to calculate and compare the proportion of households that incur catastrophic health expenditures. The logistic regression model was employed in analyzing the factors associated with catastrophic health expenditures. The concentration of catastrophic health expenditures inequality was assessed using the concentration curve, whereas the inequality size was determined using the concentration index. The decomposition method was used to decompose the concentration index into determining components.

**Results:**

Relative to the fixed threshold value, the rank-dependent threshold revealed a higher share of households facing catastrophic health expenditures i.e., from 27% in 2010/2011 to 48% in 2015/2016. The two thresholds reveal similar trend, but differ in percentage points. The key factors associated with catastrophic health expenditures were economic status and geopolitical zone. Inequality in catastrophic health expenditures was found to be concentrated among the poor. The household economic status was uncovered as the major positive contributor to catastrophic health expenditures inequality across the sample periods.

**Conclusion:**

The findings of the study imply that narrowing economic status gap across households, and increasing the depth of insurance are crucial mechanisms to reduce the probability of incurring catastrophic health expenditures among the poor in Nigeria.

## Background

Catastrophic health expenditure (CHE) widely described as health care payment beyond a certain fraction, say 10% of household income, has been well reported in health economics literature to possess negative economic consequences. These negative economic repercussions, range from sacrifice of basic goods and services, depletion of savings by individuals and families, to loss of income, and productivity, as well as disruption of welfare and living standard, particularly in developing countries where less functional health systems dominate [1]. There is indeed a pervasive agreement among health scientists and policy makers, that a well-functioning health system ensures financial protection of health service users, from catastrophic financial effects of ill health and also assures universal coverage for all individuals. This led the World Health Organization (WHO) to advocate for affordable universal coverage and equal access to health services for all citizens through its remarkable policy report captioned ‘Health systems financing: The path to universal coverage’. The major target of the WHO in this notable policy report published in 2010 is to reduce reliance on out-of-pocket (OOP) payments for health services and promote prepayment mechanisms, such as health insurance. The 2010 WHO resolution notwithstanding, Nigeria still have very poor universal health coverage (UHC) [2]. This is mainly because majority of the population still faces financial hardship as a result of seeking needed medical care. Notably, the Nigeria health financing system depends on various sources of health sector funding – government budgetary allocations, revenue collections from direct and indirect taxes, aside OOP and insurance. Tax revenues are pooled at the federal level and are distributed among the three tiers of government (federal, state and local government levels). These funds are generally inadequate, and barely complement the bulk of health spending in the form of household OOP expenditures. For instance, the total government budgetary allocation to the health sector has remained persistently low, at an average of about 5%, against the international benchmark of 15% [3]. Total expenditure from various levels of government is on average only 29% of total health spending, as compared with the private sector expenditure, as much as 70% [4]. Low funding from various tiers of government has led to inadequate health infrastructural facilities and poor access to quality health care services in Nigeria. Hence, OOP expenditures remain the dominant source of financing the country’s health system, indicating lack of financial protection. Further, there has been slow progress in coverage of essential health care services, particularly preventive health services, and this has worsened the disease burden in the country [5].

Nigeria is still far below the UHC health insurance financing target of 90%, as its health insurance contribution to total health expenditure (THE) remains at an average of only 2% [3]. To complement the NHIS and commit better to United Nations Sustainable Development Goal 3 (SDG3) and UHC, the Nigeria government in 2014 passed into law the National Health Bill. This, in principle, targets the provision of increased access to basic health care for the vulnerable population. Disappointingly, even with this law in place, many Nigerians still pay out of their pocket for medical expenses, and this has in fact continued to drive a considerable number of families to experiencing CHE [6]. About 97% of Nigerians are uncovered from financial hardship of huge medical bills and among these are the less privileged and vulnerable groups [7]. The WHO revealed in its 2010 report that a country can achieve financial protection, indicated by negligible levels of incidence of financial catastrophe when its OOP payments fall to 15–20% of THE.

However, OOP payment in Nigeria remains worrisomely high, as it proportion of total health and gross private health expenditure is still above 70 and 90% respectively [4]. More so, government health spending as a percentage of the gross domestic product (GDP) has for more than a decade remained less than 1%, implying that OOP payment is the dominant health financing system in Nigeria [4]. This excess reliance on OOP expenditure for medical bills curbs health care consumption, deepens unequal access to quality health care and exposes Nigeria households to incurring CHE, hence making the goal of UHC far from been realised [8].

Updated existing studies [e.g., 9, 10, 11, 12] on CHE incidence have only used a single period survey dataset (2009 living standard survey), which is not only dated, but different from that of the current study. Further, the studies of [13, 14; 15 among others] only used small sample state level single period datasets, clearly not nationally representative and unable to generally represent Nigeria – a country with the largest population in Africa. The current works of [16–18] are only a review of previous studies on CHE incidence in Nigeria. The study of [19] explored the determinants of CHE in Nigeria, but focused only on poorly insured elderly households in a single point in time. Omotosho and Ichoku [9] also used one single period dataset in analysing CHE correlates in Nigeria. Hence, country specific trend analysis tracking progress in addressing financial risk in terms of CHE is yet to be done with several comparable datasets in Nigeria.

To the best of author’s knowledge, recent studies on concentration of inequality in CHE, together with its decomposition are scarce in Nigeria. In Nigeria, available dated works of [20, 21] only used small sample state level primary datasets to uncover the size of inequality in CHE, without decomposing their contributing socioeconomic factors. Hence, it becomes crucial to do further investigation across multiple time periods on not just the incidence, and correlates of CHE, the magnitude of inequality in CHE in Nigeria, but its socioeconomic concentration and decomposition in the country. Such decomposition analysis could aid policy makers to track changes in the real factors that tend to push the poor Nigerian households into experiencing financial catastrophe.

In light of the above identified gaps, the current study contributes to literature in the following useful ways. First, to track Nigeria’s progress towards UHC, the study utilized several rounds of the Nigeria General Household surveys (NGHS) implemented in 2010/2011, 2012/2013 and 2015/2016 to not only do a detailed trend analyses of the proportion of Nigeria households that incur CHE, but also examined exhaustively it corresponding determining socioeconomic factors. In designing the Nigerian health system, policy makers not only need to know the changes that have occurred in the proportion of households facing CHE, but also the varying contributions of it driving socioeconomic factors across multiple time periods. Second, this paper provides fresh empirical evidence for the first time in Nigeria, on the concentration of inequality in CHE, the size of this inequality and its determining factors using the decomposition method of analysis and the three comparable panel survey datasets mentioned above. Results from achieving these objectives may be useful in enhancing the Nigeria health insurance programs to not just reduce unequal distribution of CHE, but carefully handle its key contributing factors. These results may also inform policies that will help in efficient management and utilization of resources in raising the health status level of vulnerable groups, thus draw Nigeria households closer to UHC. Third, unlike previous studies, another contribution of this paper is the documentation of not just national, but also sub-national (regional) patterns of CHE proportion, inequality concentration, determining factors and decomposition in Nigeria. Nigeria is a highly heterogeneous country, so that the issues of incidence, inequality and drivers of CHE can indeed differ across urban and rural regions.

This study, therefore, made effort to achieve five specific objectives in Nigeria: First, it examined the proportion of households that incur CHE. Second, it estimated the factors associated with CHE. Third, it determines whether inequality in CHE is pro-poor or pro-rich. Fourth, it estimated the size of inequality in CHE. Finally, it decomposed inequality in CHE into its determining socioeconomic factors. The rest of the paper is structured as follows: The next section describes the methodology and datasets. The results are presented and analysed in section 3. Section 4 reveals the discussion, while the final section is the conclusion and policy implications.

## Methods

### Data sources

Data for this study was drawn from the 2010/2011, 2012/2013 and 2015/2016 nationally representative panel NGHS. The NGHS datasets are comparable and representative of the 36 states in Nigeria and Federal Capital Territory (FCT), Abuja, with each of its waves covering around 5000 households. The survey cover a wide range of socio-economic topics, which are collected through the Household, Agriculture and Community Questionnaires. In terms of the sample size, 10 households are selected per enumeration areas (EAs), where the EAs/clusters are 500, so that the total number of household interviewed is 5000. The survey is produced by the Nigeria National Bureau of Statistics (NBS) in collaboration with Federal Ministry of Agriculture and Rural Development, and the World Bank, with financial support from the Bill and Melinda Gates Foundation [22].

### Living standard and OOP payment measurement

To measure living standard, this study used total household consumption expenditures. The choice of household consumption over income is based on the fact that it is easier to collect in household surveys and also less prone to fluctuation [23]. Further, the choice of consumption over wealth in this paper is because it is widely recognised as preferred measure of household living standard relative to wealth. For instance, studies such as Montgomery et al., [24], has found some evidence that the use of wealth index to proxy for consumption resulted in biased coefficient estimates on other variables of interest. Consumption is not only rooted in economic theory, but a more direct measure of living standard. Wealth is an indirect or proxy measure of living standard that only provides an alternative when consumption data is not available [25]. However, the household data used in this study reports detailed consumption items.

The calculation of consumption aggregate in this study is based on the method specified in [26], and also on guidelines provided in [23], on the construction of consumption aggregate. Steps which were taken involves; (i) choosing a reference period (i.e., 12 months) for all relevant consumption items, (ii) adding up the total values of different components of consumption, (iii) making adjustments for cost of living differences, (iv) making adjustment for household size and composition. The various components consist of; Food consumption component: involving aggregation of total value of (i) food purchased in the market, (ii) home-produced food, (iii) other households food items acquired as gifts and (iv) food acquired from employers as payment in-kind. Non-food consumption component: involves adding up the value of purchased non-food consumption items, comprising of education, health services, rent, electricity, housing expenses and other non-foods minus lumpy expenses (funeral and marriage expenses). It is crucial to note at this point that consumption aggregate serves as a basis for the assessment of poverty in Nigeria [27, 28]. Procedures of these computations are described in much detail in [26].

The variables of interest for the computation of OOP payments are captured in section 4 of the three waves of NGHS questionnaires. The OOP payment variable for each of the survey round was calculated as sum of money spent on hospital/health facility, medicines/drugs medical supplies, and medical related goods and service fees that are not compensated by any insurance scheme.

### Measuring households CHE incidence/proportion and intensity

Previous studies (e.g., [29, 30]) have used fixed percentage threshold in the computation of CHE. Consequently, these studies ignored the fact that households of different socioeconomic status face varying thresholds in determining CHE, as in [31]. The current study incorporates greater concern for the poor in defining CHE by following Ataguba [31] to depart from the use of fixed threshold. That is, it allows variations in threshold payment levels across individuals on the income distribution range by using rank-dependent threshold. The rank-dependent threshold upon which the CHE indices computation was based is given as;

1 $$ {Z}_{cat}^{\prime }=\omega \left(p:\gamma \right)\ast {Z}_{cat} $$

Where *p* is household’s percentile, *γ* is the parameter of aversion to inequality, *Z*_*cat*_ is the initial threshold level, and *ω*(*p* : *γ*) = *γ*(1 − *p*)^*γ* − 1^ for *γ* ∈ (0, 1). This limiting condition means that when *γ* = 1, ${Z}_{cat}^{\prime }={Z}_{cat}.$ Put differently, the catastrophic threshold becomes a constant as in previous papers. As previously stated, this study follows Ataguba [31] to use different values of *γ* (i. e., 0.8 and 1.0), as bases for computing the incidence and intensity of CHE in Nigeria. This is to allow the catastrophic threshold vary across the entire distribution and avoid underreporting of CHE cases. Following standard literature, the CHE incidence, intensity, and gap were determined in terms of rank-dependent CHE incidence which measures the proportion of sampled households whose OOP health expenditure as a fraction of their total expenditure exceeds the specified threshold, the CHE rank-dependent overshoot which indicates the average degree by which health payment (as fraction of expenditure) exceed a given threshold, and rank-dependent CHE overshoot measuring the average proportion of household rank-dependent CHE intensity in terms of the size of incidence.

### Measuring factors associated with CHE

The study employed logistic regression model to examine the factors associated with CHE in Nigeria.

2 $$ CHE=\alpha +\beta X+{\mu}_i $$

Where; *CHE* is catastrophic health expenditure, which takes 1 if household incurs CHE and 0 otherwise. The vector of explanatory variable (*X*) comprises of economic status, geopolitical zone, region of residence**,** size of the household**,** sex of household head**,** education status of household head, employment status of household head, insurance status of household, and presence of elders in the household.

### Measuring pro-poor/pro-rich inequality in CHE

To determine whether inequality in CHE in Nigeria is concentrated among the poor or rich, the study used the concentration curve. The concentration curve can be used to assess whether or not a health variable is more unequally distributed to the disadvantage of the poor. It plots the cumulative percentage of the health variable (CHE on the y-axis) against the cumulative percentage of the population, ranked by living standard, starting with the poorest, and ending with the richest (x-axis). If every individual, irrespective of his or her income, has exactly the same value of the health variable, the concentration curve will be a 45-degree line, running from the bottom left-hand corner to the top right-hand corner. If, by contrast, the health variable takes higher (lower) values among poorer households, the concentration curve will lie above (below) the line of equality. The farther the curve is above the line of equality, the more concentrated the health variable is among the poor. The reverse is the case, the farther the curve is below the line of equality [25].

### Measuring the size of CHE inequality

To estimate the size of inequality in CHE, the concentration index was computed. Concentration curve can be used to identify whether socioeconomic inequality in a health variable exists, but it does not give a measure of the size of inequality which can be used for convenient comparison. The concentration index enables the estimation of the size of socioeconomic related inequality in a health variable [25]. The index is defined as twice the area between the concentration curve and the line of equality. If there is no socioeconomic-related inequality, the concentration index is zero. Conventionally, the index takes a negative value when the curve lies above the line of equality, indicating disproportionate concentration of the health variable among the poor, and a positive value when it lies below the line of equality. It can be obtained by the formula;

3 $$ C=\frac{2}{N\mu}\ \sum \limits_{i=1}^n{h}_i{r}_i-1 $$

Where *C* is the concentration index, *h*_*i*_ is the health sector variable, *μ* is its mean, *r*_*i*_ is the fractional rank of the individual in the socioeconomic distribution with *i* = 1 for the poorest and *i* = N for the richest. It is important to note that the concentration of CHE in this paper has been calculated using the Stata package conindex which accounts for bounded limits. This is so since the CHE is a binary variable that takes 1 or 0.

### Decomposition of CHE concentration index

The decomposition method proposed by Wagstaff et al., [32] was employed in examining the factors that contribute to inequality in CHE. The advantage of this method over linear and non-linear regression models is that it allows one to estimate the relative contribution of factors to inequality in a health variable. For a linear additive regression model, the CHE variable *Y*_*i*_ is represented in terms of the intercept *α*, the relative contribution of *X*_*ki*_ factors and error term *ε*_*i*_ in Eq. (4) below;

4 $$ {Y}_i=\alpha +{\sum}_k{\beta}_k{X}_{ki}+{\varepsilon}_i $$

Based on Eq. (4) above, the CHE concentration index can be decomposed as follows;

5 $$ C=\sum \limits_k\left(\frac{\beta_k{\overline{X}}_k}{\mu}\right){C}_k+\frac{GC_{\varepsilon }}{\mu } $$

In Eq. (5), the value *β*_*k*_ denotes the regression coefficient of CHE variable on determinant *X*_*k*_, ${\overline{X}}_k$ is the mean of *X*_*k*_, *C*_*k*_ is the concentration index of *X*_*k*_, and *GC*_*ε*_ represents the generalised concentration index for the error term (*ε*). As recorded above, CHE is measured as a binary variable. Following from this, the standard logit is preferred for the estimation of CHE. However, since the logit is intrinsically non-linear, as opposed to the decomposition model which is basically linear, the study used the natural logarithm of the odds of the CHE in the decomposition model instead of the observed CHE, as in [33].

## Results

### Proportion of household CHE incidence and intensity

Tables 1, 2 and 3 showcase the results of catastrophic indices using total household consumption expenditure at national and regional levels. The current paper considered various initial thresholds of 10, 15 and 20%, together with varying parameter values (0.8 and 1.0) as suggested in [31]. For each of the initial threshold, the rank-dependent catastrophic headcount changes with the value of the parameter. Decreasing the value of the parameter, increases the headcount CHE for all thresholds, and across national and regional areas in all periods of investigation. For instance, at 10% threshold, the headcount increased from about 23 to 27%, 18 to 22%, and 43 to 48%, when the parameter value was decreased from 1.0 to 0.8 in 2010/2011, 2012/2013 and 2015/2016 respectively. These are the national level estimates, as seen in Table 1. Though, a similar trend is observed at the regional level, percentage point estimates differ between urban and rural regions of the country. Relative to headcount CHE estimates in the urban region, those of the rural region appear to be higher. As in Table 2, the headcount CHE in urban region is roughly 24, 21 and 39% in 2010/2011, 2012/2013 and 2015/2016 respectively, at 10% threshold and 0.8 parameter value. However, as in Table 3 the rural region estimates are relatively higher, yielding 27, 23 and 51% in 2010/2011, 2012/2013 and 2015/2016 respectively. This is attributable to the fact that the households in rural areas make more OOP than households in urban areas. Just in line with the finding that on average OOP in urban areas was 13% whereas it was 17% in rural areas in 2010–2016 period. Hence, households in rural areas tend to face a higher catastrophic burden of medical payments, relative to those residing in urban areas.

**Table 1:** Catastrophic out-of-pocket health-care payment (total household expenditure) indices in Nigeria

	Wave 1 (2010/2011)						Wave 2 (2012/2013)						Wave 3 (2015/2016)					
	Threshold = 10%		Threshold = 15%		Threshold = 20%		Threshold = 10%		Threshold = 15%		Threshold = 20%		Threshold = 10%		Threshold = 15%		Threshold = 20%	
	*γ* = 0.8	*γ* = 1.0	*γ* = 0.8	*γ* = 1.0	*γ* = 0.8	*γ* = 1.0	*γ* = 0.8	*γ* = 1.0	*γ* = 0.8	*γ* = 1.0	*γ* = 0.8	*γ* = 1.0	*γ* = 0.8	*γ* = 1.0	*γ* = 0.8	*γ* = 1.0	*γ* = 0.8	*γ* = 1.0
Headcount measure																		
*H*′_*cat*_	26.55	22.70	18.59	15.28	13.96	10.94	22.18	18.24	15.07	12.16	11.02	8.89	47.64	42.88	36.85	32.04	29.52	25.44
Gap measures																		
*G*′_*cat*_	5.71	5.27	4.80	4.34	4.15	3.69	5.27	4.80	4.53	4.15	4.00	3.63	18.95	18.14	17.22	16.20	15.87	14.86
*MPG*′_*cat*_	21.50	23.20	25.85	28.41	29.72	33.78	23.74	26.85	30.03	34.14	36.20	40.88	39.77	42.31	46.75	50.85	53.76	58.44

Note: All estimations are appropriately weighted to be nationally representative

**Table 2:** Catastrophic out-of-pocket health-care payment (total household expenditure) indices in Urban Region

	Wave 1 (2010/2011)						Wave 2 (2012/2013)						Wave 3 (2015/2016)					
	Threshold = 10%		Threshold = 15%		Threshold = 20%		Threshold = 10%		Threshold = 15%		Threshold = 20%		Threshold = 10%		Threshold = 15%		Threshold = 20%	
	*γ* = 0.8	*γ* = 1.0	*γ* = 0.8	*γ* = 1.0	*γ* = 0.8	*γ* = 1.0	*γ* = 0.8	*γ* = 1.0	*γ* = 0.8	*γ* = 1.0	*γ* = 0.8	*γ* = 1.0	*γ* = 0.8	*γ* = 1.0	*γ* = 0.8	*γ* = 1.0	*γ* = 0.8	*γ* = 1.0
Headcount measure																		
*H*′_*cat*_	24.38	20.50	16.97	13.76	12.12	9.55	21.17	17.83	14.03	10.99	9.82	8.30	39.11	33.82	28.83	23.50	21.35	17.68
Gap measures																		
*G*′_*cat*_	5.19	4.78	4.37	3.94	3.78	3.38	3.98	3.62	3.27	2.93	2.79	2.46	11.92	11.26	10.55	9.83	9.54	8.83
*MPG*′_*cat*_	21.31	23.34	25.75	28.68	31.23	35.38	18.79	20.35	23.31	26.67	28.43	29.64	30.48	33.28	36.60	41.85	44.69	49.93

**Table 3:** Catastrophic out-of-pocket health-care payment (total household expenditure) indices in Rural Region

	Wave 1 (2010/2011)						Wave 2 (2012/2013)						Wave 3 (2015/2016)					
	Threshold = 10%		Threshold = 15%		Threshold = 20%		Threshold = 10%		Threshold = 15%		Threshold = 20%		Threshold = 10%		Threshold = 15%		Threshold = 20%	
	*γ* = 0.8	*γ* = 1.0	*γ* = 0.8	*γ* = 1.0	*γ* = 0.8	*γ* = 1.0	*γ* = 0.8	*γ* = 1.0	*γ* = 0.8	*γ* = 1.0	*γ* = 0.8	*γ* = 1.0	*γ* = 0.8	*γ* = 1.0	*γ* = 0.8	*γ* = 1.0	*γ* = 0.8	*γ* = 1.0
Headcount measure																		
*H*′_*cat*_	27.46	23.61	19.32	15.90	14.73	11.51	22.59	18.39	15.49	12.60	11.50	9.10	50.71	46.16	39.79	35.14	32.49	28.25
Gap measures																		
*G*′_*cat*_	5.92	5.46	4.98	4.50	4.30	3.82	5.75	5.37	5.00	4.61	4.46	4.07	21.49	20.64	19.64	18.63	18.16	17.05
*MPG*′_*cat*_	21.57	23.15	25.81	28.30	29.19	33.21	25.47	29.24	32.29	36.61	38.76	44.77	42.38	44.71	49.36	53.03	55.90	60.73

Results for the rank-dependent catastrophic gap measures follow a trend similar to that of the rank-dependent catastrophic headcount measures, for all initial threshold and across national and regional areas in periods of time considered in this paper. At 10% initial threshold for example, the catastrophic gap (${G}_{cat}^{\prime }$) increased from about 5.3% (for *γ* = 1.0) to 5.7% (for *γ* = 0.8), 4.8% (for *γ* = 1.0) to 5.3% (for *γ* = 0.8), and 18.1% (for *γ* = 1.0) to 18.9% (for *γ* = 0.8) in 2010/2011, 2012/2013 and 2015/2016 respectively. These are the national estimates in Table 1. As previously mentioned, though a similar trend is seen at the regional levels, percentage points vary across urban and rural regions. At 10% threshold and 0.8 parameter value, catastrophic gap in urban region was 5.2, 3.9 and 11.9%, whereas those of the rural region were 5.9, 5.8 and 21.5% in 2010/2011, 2012/2013 and 2015/2016 respectively. The reason for differences being the higher concentration of burden of OOPs at the rural, relative to the urban region as already stated above. In addition, across the three periods of time, the mean positive gap $\left({MPG}_{cat}^{\prime}\right)$ persistently decreased as the value of the parameter for each threshold decreased at both national and regional levels.

Of importance to note is that results across thresholds conform to a priori rationale at both national and regional levels. This is because the rank-dependent headcount decreased for higher thresholds. For instance, as seen in national estimates in Table 1, for the value of the parameter *γ* = 0.8, the headcount index in 2010/2011 decreased from 26.6% (*Z*_*cat*_ = 10%) to 18.6% (*Z*_*cat*_ = 15%) and to 13.0% (*Z*_*cat*_ = 20%). In 2012/2013, the index decreased from 22.2% (*Z*_*cat*_ = 10%) to 15.1% (*Z*_*cat*_ = 15%) and to 11.0% (*Z*_*cat*_ = 20%) whereas in 2015/2016, the index decreased from 47.6% (*Z*_*cat*_ = 10%) to 36.9% (*Z*_*cat*_ = 15%) and to 29.5% (*Z*_*cat*_ = 20%). In terms of the gap measures, results follow similar direction also across national and regional levels, the rank-dependent catastrophic gaps decrease as initial thresholds increase, whereas the mean positive gap increase with increasing initial threshold values.

Comparing the results gotten using a rank-dependent threshold with those based on fixed threshold used in [29] for a normal threshold of say 10% of total household expenditure (see Tables 1, 2 and 3 for both national and regional results), the catastrophic payment headcount and gaps increased with decreasing parameter values. From the above stated, employing a fixed threshold value reduces the catastrophic headcount as compared with rank-dependent threshold which varies across the whole income distribution ladder by decreasing the thresholds for the poor. Put differently, the fixed threshold value understates the number of households that experience CHE, as compared to the rank-dependent threshold. These results vary across regions, since the OOP burden is more on the rural than the urban households in Nigeria.

### Average trends in household OOP composition, % Total expenditure

Household OOP payment across national and regional areas, shown in Table 4 varied across different medical expenditure components in all periods of time. At the national level, money spent on drugs rose from 4% in 2010 to 10% in 2016. While these estimates roughly remained the same in the urban region, they tend to be higher in the rural region. As seen in Table 4, the amount of money spent on drugs in the urban region was roughly 4 and 10% in 2010 and 2016. However, in the rural region, these estimates rose to 7 and 11% in 2010 and 2016 respectively. This means households in rural areas spent more money in the purchase of over the counter drugs, relative to households in urban areas. This result is of no surprise since poor people usually in rural areas always prefer cheap self-medication whenever they fall ill, relative to costly hospital treatment. More so, households spent more money on hospital facilities in 2016 (about 3%), as compared to money spent in 2010 (only 1%). On average, these estimates seem not to differ significantly across urban and rural areas in the country. Similar trend is also applicable to money spent on medical services, with about 5% increase at national level, and roughly 7% increase at regional levels in 2010–2016 periods. No reasonable change, however, was observed in amount of money spent on medicines and medical supplies within the study period at both national and regional levels. Summarily, total OOP payments on health care doubled within study period, rising from roughly 10% in 2010 to 22% in 2016. The doubling of the OOP payments within the study period is mainly driven by the relatively large increase in money spent on drugs/pharmaceuticals by Nigerian households, from only 4% in 2010 to 10% in 2016. Nowadays, poor households would first seek over the counter drugs immediately they fall sick, since going to the hospital to see the doctor is relatively costly.

**Table 4:** Average Trends in Household OOP Composition, % Total Expenditure, both National and Regional Estimates, 2010–2016

		National Estimates			Urban Estimates			Rural Estimates		
n/s	OOP component expenses	2010/2011	2012/2013	2015/2016	2010/2011	2012/2013	2015/2016	2010/2011	2012/2013	2015/2016
1	Over the counter drugs	0.04	0.03	0.10	0.04	0.03	0.10	0.07	0.04	0.11
2	Hospital facility	0.01	0.004	0.03	0.003	0.003	0.02	0.01	0.004	0.03
3	Medicines and medical supplies	0.01	0.01	0.01	0.01	0.01	0.03	0.02	0.01	0.03
4	Medical services	0.03	0.04	0.08	0.03	0.03	0.11	0.05	0.03	0.10
5	Total OOP Payments	0.10	0.08	0.22	0.083	0.073	0.26	0.15	0.084	0.27

### Associated factors of CHE

Tables 5, 6 and 7 presents the estimated odd ratios with corresponding robust standard errors, and 95% confidence intervals. These odd ratios were obtained from the logistic regression analyses for the three rounds of the NGHS using 10% of total household expenditure across national and regional levels. As anticipated, households in the richest quintile has lesser odds of incurring CHE, as compared to households in the poorest quintile. This result is observed both at the national level and across urban and rural regions. At the national level shown in Table 5, the odds of incurring CHE for households in the richest quintile are roughly only 0.05, 0.18, and 0.03, relative to the higher odds in the poorest quintiles in 2010/2011, 2012/2013 and 2015/2016 respectively. A similar trend is observed across urban and rural regions. In urban and rural regions, the odds of CHE tend to be higher for households in poor quintiles, relative to households in rich quintiles. At the national level, households in the southern zone are 4.5 times more likely to incur CHE in 2015/2016, relative to households in the northern zone.

**Table 5:** Logistic Regression of Factors Associated with CHE in Nigeria

Variables	Wave 1 (2010/2011)			Wave 2 (2012/2013)			Wave 3 (2015/2016)		
	Odds Ratio	Robust Std. Err	95% Confidence Interval	Odds Ratio	Robust Std. Err	95% Confidence Interval	Odds Ratio	Robust Std. Err	95% Confidence Interval
**Economic Status**									
Poorest (Ref)									
Second	0.7687	0.3933	0.2820–2.0953	0.5799	0.3032	0.2081–1.6158	0.3769^c^	0.1047	0.2186–0.6498
Middle	0.0000	0.0000	0.0000–0.0000	0.3818	0.2234	0.1213–1.2020	0.3594^c^	0.0954	0.2136–0.6047
Fourth	0.2389^b^	0.1459	0.0722–0.7905	0.2311^b^	0.1625	0.0583–0.9168	0.1270^c^	0.0521	0.0568–0.2839
Richest	0.0469^c^	0.0555	0.0046–0.4784	0.1770^b^	0.1403	0.0374–0.8373	0.0347^c^	0.0222	0.0099–0.1215
**Zone**									
North (Ref)									
South	1.6778	1.4867	1.2955–9.5276	0.4524	0.2957	0.1256–1.6290	4.4655^c^	1.8566	1.9768–10.0871
**Sector**									
Rural (Ref)									
Urban	1.5896	0.7293	0.6468–3.9067	0.4773	0.2344	0.1823–1.2496	0.8687	0.2316	0.5152–1.4647
**HHsize**									
Less than 5 members (Ref)									
Greater than 5 members	0.7960	0.3439	0.3421–1.8568	0.4882^a^	0.2026	0.2165–1.1000	1.0456	0.3831	0.5098–2.1443
**Gender**									
Female (Ref)									
Male	1.3880	0.6487	0.5561–3.4694	1.1173	0.4494	0.5079–2.4578	0.7777	0.1696	0.5071–1.1927
**Education**									
No (Ref)									
Nursery & primary	0.5678	0.3293	0.1822–1.7697	1.9085	0.6136	0.2418–3.4137	0.9790	1.1011	0.1080–8.8744
Secondary	0.5456	0.4635	0.1032–2.8830	0.7403	0.4775	0.2091–2.6206	1.0819	1.2112	0.1206–9.7077
Post-secondary	1.6935	1.0446	0.5055–5.6735	0.0000	0.0000	0.0000–0.0000	2.1435	2.4483	0.2285–20.1063
**Employment status**									
Employed (Ref)									
Unemployed	0.5165	0.3024	0.1639–1.6273	2.2102^a^	1.0623	0.8616–5.6697	2.6685^c^	0.6930	1.6029–4.4425
**Bednet**									
No (Ref)									
Yes	0.3017^a^	0.2120	0.0761–1.1961	0.7502	0.3543	0.2973–1.8932	1.2770	0.3673	0.7275–2.2449
**Insurance**									
Uninsured (Ref)									
Insured	0.8025	1.2576	0.0372–17.3123	3.5336	3.2179	0.5930–21.0554	0.4272	0.4953	0.0440–4.1447
**Elderly**									
No (Ref)									
Yes	3.0294^b^	1.6891	1.0157–9.0357	0.6973	0.4002	0.2264–2.1478	1.0161	0.2437	0.7027–1.7157
Cons	0.0472	0.0324	0.0123–0.1815	0.0921	0.0581	0.0268–0.3170	0.0937	0.1098	0.0097–1.1214

^a^, ^b^, and ^c^ indicate significance at 10, 5 and 1% respectively

**Table 6:** Logistic Regression of Factors Associated with CHE in Urban Region

Variables	Wave 1 (2010/2011)			Wave 2 (2012/2013)			Wave 3 (2015/2016)		
	Odds Ratio	Robust Std. Err	95% Confidence Interval	Odds Ratio	Robust Std. Err	95% Confidence Interval	Odds Ratio	Robust Std. Err	95% Confidence Interval
**Economic Status**									
Poorest (Ref)									
Second	0.1309^a^	0.1032	0.0279–0.6138	0.5787^a^	0.1644	0.3315–1.0101	0.5116	0.3607	0.1284–2.0376
Middle	0.2207^a^	0.1761	0.0461–1.0549	0.3312^c^	0.0931	0.1908–0.5748	0.1866^c^	0.1028	0.0634–0.5493
Fourth	0.0489^c^	0.0365	0.0113–0.2117	0.4504^b^	0.1322	0.2534–0.8008	0.1287^c^	0.0669	0.0464–0.3569
Richest	0.0183^c^	0.0124	0.0048–0.0691	0.1243^c^	0.0374	0.0688–0.2244	0.0389^c^	0.0196	0.0144–0.1048
**HHsize**									
Less than 5 members (Ref)									
Greater than 5 members	0.7080	0.2462	0.35815–1.3997	0.7461	0.1346	0.5238–1.0628	1.6918	0.6530	0.7939–3.6050
**Gender**									
Female (Ref)									
Male	0.5784	0.2252	0.2696–1.2407	1.1299	0.1953	0.8052–1.5856	0.9733	0.2220	0.6223–1.5220
**Education**									
No (Ref)									
Nursery & primary	1.6406	0.6026	0.7986–3.3704	1.3124	1.3135	0.1845–9.3322	0.3000	0.3465	0.0311–2.8862
Secondary	0.0000	0.0000	0.0000–0.0000	0.9502	0.9504	0.1337–6.7490	0.6208	0.7185	0.0642–5.9996
Post-secondary	4.8863^a^	4.1511	0.9243–25.8291	0.6015	0.6033	0.0842–4.2959	0.3913	0.4587	0.0393–3.8931
**Employment status**									
Employed (Ref)									
Unemployed	0.9732	0.5646	0.3121–3.0341	0.6093^b^	0.1351	0.3945–0.9411	0.8792	0.2140	0.5455–1.4168
**Bednet**									
No (Ref)									
Yes	0.9933	0.4664	0.3957–2.4935	0.9936	0.1902	0.6826–1.4461	0.4884^c^	0.1208	0.3007–0.7933
**Insurance**									
Uninsured (Ref)									
Insured	2.0574	0.9714	0.8154–5.1909	13.0746^b^	9.5684	3.1152–54.8742	8.6332^b^	9.4220	1.0167–73.3076
**Elderly**									
No (Ref)									
Yes	0.7632	0.3348	0.3230–1.8033	0.6656^a^	0.1425	0.4375–1.0126	0.5677^b^	0.1279	0.3650–0.8829
Cons	74.0551	53.9061	17.780–308.433	4.9146	5.0138	0.6654–36.2964	189.7624	238.1426	16.2178–2220.37

^a^, ^b^, and ^c^ indicate significance at 10, 5 and 1% respectively

**Table 7:** Logistic Regression of Factors Associated with CHE in Rural Region

Variables	Wave 1 (2010/2011)			Wave 2 (2012/2013)			Wave 3 (2015/2016)		
	Odds Ratio	Robust Std. Err	95% Confidence Interval	Odds Ratio	Robust Std. Err	95% Confidence Interval	Odds Ratio	Robust Std. Err	95% Confidence Interval
**Economic Status**									
Poorest (Ref)									
Second	0.6180	0.4020	0.1727–2.2116	0.2824^c^	0.0672	0.1771–0.4503	0.1981^b^	0.1418	0.0487–0.8062
Middle	0.1593^c^	0.0971	0.0482–0.5261	0.1317^c^	0.0297	0.0846–0.2052	0.0885^c^	0.0583	0.0243–0.3218
Fourth	0.0892^c^	0.0529	0.02787–0.2857	0.0896^c^	0.0205	0.0573–0.1403	0.0381^c^	0.0238	0.0111–0.1302
Richest	0.0473^c^	0.0282	0.0146–0.1525	0.0437^c^	0.0104	0.0274–0.0697	0.0090^c^	0.0055	0.0027–0.0300
**HHsize**									
Less than 5 members (Ref)									
Greater than 5 members	0.6736^a^	0.1361	0.4533–1.0011	0.9527	0.1161	0.7503–1.2099	2.5039^c^	0.7638	1.3770–4.5527
**Gender**									
Female (Ref)									
Male	1.0528	0.2055	0.7181–1.5436	0.8590	0.1003	0.6833–1.0800	1.0136	0.1751	0.7224–1.4221
**Education**									
No (Ref)									
Nursery & primary	1.4978^a^	0.3556	0.9405–2.3854	1.8219	0.8585	0.7234–4.5881	0.8993	0.7120	0.1905–4.2450
Secondary	1.4262	0.4703	0.7472–2.7222	2.4452^a^	1.1593	0.9655–6.1927	0.8314	0.6579	0.1762–3.9214
Post-secondary	0.8273	0.3091	0.3977–1.7209	1.7189	0.8395	0.6599–4.4771	0.8308	0.6774	0.1680–4.1077
**Employment status**									
Employed (Ref)									
Unemployed	1.2218	0.3098	0.7433–2.0085	0.8569	0.1301	0.6363–1.1539	0.6403^b^	0.1385	0.4190–0.9786
**Bednet**									
No (Ref)									
Yes	1.4951^a^	0.3442	0.9520–2.3478	1.3276^b^	0.1681	1.0359–1.7016	0.4477^c^	0.0774	0.3189–0.6286
**Insurance**									
Uninsured (Ref)									
Insured	4.5179	5.1117	0.4918–41.4976	8.2673^c^	5.9806	2.0026–34.1292	0.2254^b^	0.1435	0.0647–0.7851
**Elderly**									
No (Ref)									
Yes	1.1895	0.3006	0.7248–1.9520	0.7724^a^	0.1176	0.5730–1.0410	0.7246^a^	0.1264	0.5147–1.0201
Cons	28.1258	17.6393	8.2275 96.1488	6.6019	3.4335	2.3822–18.2962	211.7662	264.0777	18.3815–2439.64

^a^, ^b^, and ^c^ indicate significance at 10, 5 and 1% respectively

Furthermore, households larger than 5 members have low odds (0.5) of experiencing CHE, as compared to households less than 5 members in 2012/2013. This result tend to differ across regional dimensions, as in Tables 6 and 7. Larger households in rural regions tend to have higher odds (2.5) of incurring CHE, compared to the odds (1.7) of larger households in urban regions. At the national level, unemployed household heads have 2.2 times and 2.7 times higher odds of facing CHE, relative to employed household heads in 2012/2013 and 2015/2016 respectively. Unemployed household heads in rural region have a more significant odds of CHE, compared to unemployed household heads with insignificant odds in the urban region in 2015/2016. In terms of insurance status, results for the insured households were found statistically insignificant at the national level. At the regional level, insurance barely reduces the odds of experiencing CHE. Nationally, households with elderly members have roughly 3.0 times increased odds of incurring CHE, comparative to households without elderly members in 2010/2011. The odds of incurring CHE for the elderly are higher in rural regions, relative to the odds faced by the elderly in urban regions in 2015/2016. This result is of no surprise since elderly household members tend to concentrate more in the rural than in the urban regions.

### Measurement of pro-poor/pro-rich inequality in CHE

**Fig. 1:**
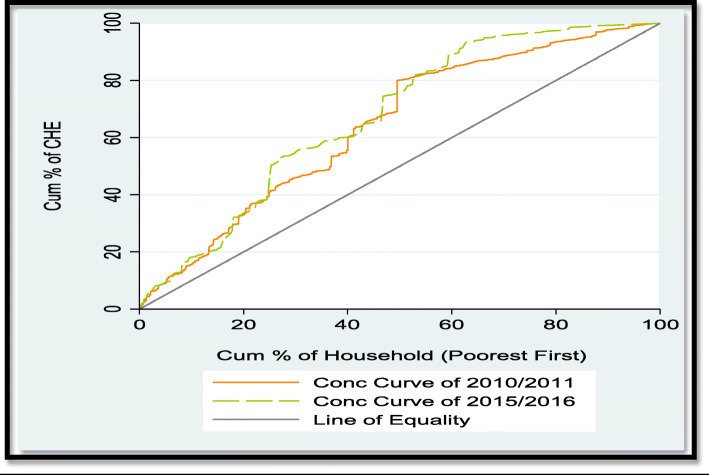
Concentration curves of CHE in Nigeria

Figure 1 is a pictorial analyses of the socioeconomic ladder upon which inequality in CHE concentrates. This was computed using 10% of total household expenditure. It provides the concentration curve for two extreme data rounds (i.e., 2010/2011 and 2015/2016), with the aim to carry out a dominance testing on the change in CHE inequality between the two extreme study samples, as done in related previous papers [34, 35]. First, to make inference on dominance between the concentration curves and the line of equality, the study employed the multiple comparison approach (mcp). This approach, which rejects the null of non-dominance in favour of dominance if there is at least one significant difference between curves in one direction and no significant difference in the other, reveals that the CHE concentration curve dominates the line of equality in 2010/2011 and 2015/2016, and this result is significant at 5% level (see Table 8). This implies that the concentration curves of the two periods are above the 45° line, indicating that the CHE inequality concentrates among the poor. Second, to test for dominance between 2010/2011 and 2015/2016 CHE concentration curves, the current paper utilized the intersection union principle (iup) method. The iup result, which requires significant difference between ordinates at all quantile points to accept dominance, strongly confirm the result of mcp in showcasing that there is statistically insignificant dominance of one curve against the other (see Table 9). This simply suggests that there is no significant change in CHE inequality between 2010/2011 and 2015/2016 (Tables 8 and 9).

**Table 8:** Test of Dominance between CHE Concentration Curves and the Line of Equality

2010/2011				2015/2016			
Variable	Sign Level	No of Points	Rule	Variable	Sign Level	No of Points	Rule
CHE	5%	19	mca	CHE	5%	19	mca
Result	Concentration Curve Dominates			Result	Concentration Curve Dominates

**Table 9:** Test of Dominance between 2010/2011 and 2015/2016 CHE Concentration Curves

Data 1	Data 2	Sign Level	No of Points	Rule	Data 1	Data 2	Sign Level	No of Points	Rule
2010/2011	2015/2016	5%	19	mca	2010/2011	2015/2016	5%	19	iup
Non-dominance					Non-dominance				

### The size of CHE inequality

Table 10 presents the CHE concentration indices, with their corresponding standard errors for the three data rounds, using 10% of total household expenditure at both the national and regional levels. At the national level, the CHE concentration index which was − 0.2153 in 2010/2011 fell to − 0.1658 in 2012/2013, and rose again to − 0.2042 in 2015/2016, in absolute terms. This implies that there is no significant change in CHE inequality between 2010/2011 and 2015/2016. This trend do not differ as much at the regional levels. In urban and rural regions, the CHE inequality tend to fall in 2012/2013, but rose again in 2015/2016. Notably, the sizes of CHE inequality tend to be higher in rural, relative to the urban region. This implies that CHE is more unequally distributed in the rural, compared to the urban region. More so, negative concentration indices in the three periods demonstrate that households with low economic status had higher probabilities of incurring CHE than the rich, both nationally and across regions in the country.

**Table 10:** Concentration Indices of CHE in Nigeria, both National and Regional Estimates, 2010–2016

2010/2011		National Estimates				Urban Estimates						Rural Estimates					
		2012/2013		2015/2016		2010/2011		2012/2013		2015/2016		2010/2011		2012/2013		2015/2016	
Index	Std error	Index	Std error	Index	Std error	Index	Std error	Index	Std error	Index	Std error	Index	Std error	Index	Std error	Index	Std error
−0.2153	0.0716	−0.1658	0.0853	− 0.2042	0.0465	− 0.1801	0.0922	− 0.0848	0.0171	− 0.2025	0.0853	− 0.1927	0.1149	− 0.1482	0.0105	−0.2065	0.0544

### Decomposition of CHE concentration index

To understand the observed inequalities in a binary health variable (CHE), the current study used the non-linear decomposition analyses (i.e., decomposition based on the logit model), as in the works of [33, 34]. The decomposition was estimated in terms of logit coefficients, the means of the *Ln Odds*_*CHE*_ and its corresponding determinants, elasticity of *Ln Odds*_*CHE*_, concentration index (CI) values of explanatory variables, the additively decomposed CI of the *Ln Odds*_*CHE*_, and the grouped % contribution to CI. These were done for each of the three data rounds, using 10% of total household expenditure at both national and regional levels. Results at national level in Table 11 reveal that households in the second to the poorest wealth quintile position, household in the urban area, large household size, education, and employment status of the household head are all negatively associated with economic status rank, implying concentration against the poor. On the contrary, household in the richest wealth quintile position, geopolitical zone, and gender of household head for instance, have positive association with economic status, suggesting concentration of inequality against the wealthy in 2010/2011. In 2012/2013, household in the second to the poorest wealth quintile position, being a male household head, secondary education status, insurance status, and presence of elderly members have negative concentration indices, implying concentration against the poor. Contrariwise, household in the richest wealth quintile position, household in the south, urban area, and with members greater than five for instance, have positive indices, suggesting inequality concentration against the wealthy. Results across regions, as seen in Tables 12 and 13 tend to follow similar direction with some differences. For instance, being hospitalized for ill health reduces CHE concentration against the poor in the rural region, relative to results observed in the urban region. Employment status is positively associated with economic status in rural regions across all periods of investigation, unlike in the urban regions. This means that being employed helps to reduce CHE concentration more in the rural than in the urban regions.

**Table 11:** Decomposition Analyses of CHE Concentration Index in Nigeria

Variables	Wave 1 (2010/2011)						Wave 2 (2012/2013)						Wave 3 (2015/2016)					
	Coefficient	Mean	Elasticity	Concentration Index (C)	Contribution to C	Contribution to C (%)	Coefficient	Mean	Elasticity	Concentration Index (C)	Contribution to C	Contribution to C (%)	Coefficient	Mean	Elasticity	Concentration Index (C)	Contribution to C	Contribution to C (%)
**Economic Status**																		
Poorest																		
Second	−0.0110	0.2037	−0.0914	− 0.3234	0.0295		− 0.0199	0.1945	− 0.1705	− 0.2978	0.0508		− 0.0713	0.1986	− 0.2654	− 0.3845	0.1020	
Middle	− 0.0519	0.1924	− 0.4055	0.1043	− 0.0423		− 0.0294	0.2020	− 0.2611	0.0565	− 0.0147		− 0.0716	0.1980	− 0.2669	0.0177	− 0.0047	
Fourth	− 0.0388	0.2023	− 0.3184	0.4070	− 0.1296		− 0.0348	0.2058	− 0.3151	0.4202	− 0.1324		− 0.1070	0.1979	− 0.3968	0.4605	− 0.1827	
Richest	− 0.0503	0.1933	− 0.3949	0.7560	− 0.2886	80%	− 0.0366	0.1992	− 0.3201	0.7476	− 0.2393	79%	− 0.1246	0.2036	− 0.4740	0.7127	−0.3385	92%
**Zone**																		
North																		
South	0.0189	0.4555	0.3500	0.0160	0.0056	−3%	− 0.0112	0.4455	− 0.2196	0.0110	− 0.0024	1%	0.0741	0.4380	0.6084	0.0239	0.0145	−7%
**Sector**																		
Rural																		
Urban	0.0122	0.2948	0.1461	−0.0334	−0.0049	2%	− 0.0149	0.2755	− 0.1802	0.1299	− 0.0334	14%	− 0.0070	0.2665	− 0.0131	0.0742	− 0.0026	1%
**HHsize**																		
< 5 members																		
> 5 members	−0.0044	0.4586	−0.0823	− 0.0078	0.0006	− 0%	−0.0161	0.6447	−0.4560	0.0350	−0.0164	9%	0.0022	0.8977	0.0386	0.0040	0.0002	−0%
**Gender**																		
Female																		
Male	0.0070	0.4917	0.1398	0.0119	0.0017	−1%	0.0034	0.4834	0.0712	−0.0041	−0.0003	0%	−0.0101	0.4790	−0.0908	−0.0122	0.0011	− 0%
**Education**																		
No																		
Nursery & primary	−0.0125	0.1939	−0.0985	−0.0472	0.0047		0.0216	0.4564	0.4323	0.0254	0.0109		−0.0016	0.4445	−0.0135	0.0023	−0.0000	
Secondary	−0.0139	0.0738	−0.0416	−0.0476	0.0010		0.0067	0.3873	0.2835	−0.0371	− 0.0059		0.0027	0.3649	0.0191	0.0056	0.0001	
Post-secondary	0.0135	0.1310	0.0723	−0.0577	− 0.0042	− 0%	0.0261	0.1462	0.1677	0.0197	0.0033	− 0%	0.0402	0.1729	0.1304	0.0106	0.0014	0%
**Employment status**																		
Unemployed																		
Employed	− 0.0113	0.2116	− 0.0978	− 0.0395	0.0039	−2%	0.0225	0.2053	0.2033	0.0153	0.0031	−2%	0.0556	0.2027	0.2112	0.0590	0.0127	−6%
**Bednet**																		
No																		
Yes	− 0.0182	0.3616	−0.2674	0.0040	− 0.0011	0.5%	− 0.0067	0.3102	− 0.0909	− 0.0269	0.0024	−1%	0.0118	0.2595	0.0573	− 0.0255	− 0.0024	1%
**Hospitalized**																		
No																		
Yes	−0.0162	0.0247	−0.0162	0.0136	− 0.0002	0.1%	− 0.0175	0.0230	− 0.0184	− 0.1251	0.0023	−1%	0.0176	0.0191	0.0062	0.1055	0.0007	− 0%
**Insurance**																		
Uninsured																		
Insured	−0.0201	0.0567	−0.0463	0.3220	−0.0149	7%	0.0254	0.0248	0.0277	−0.2878	− 0.0070	5%	− 0.0390	0.0092	− 0.0067	0.0242	− 0.0002	0%
**Elderly**																		
No																		
Yes	0.0324	0.1336	0.1758	0.0283	0.0040	−2%	− 0.0068	0.1790	− 0.0532	− 0.0405	0.0021	−1%	0.0057	0.3450	0.0371	0.0170	0.0007	− 0%
CI Ln Odds of CHE					−0.4348						−0.3769						−0.3977	
Mean of Ln Odds of CHE		0.0246						0.0228						0.0534				

At the national level, results of 2015/2016 is somewhat similar to that of 2012/2013. In 2015/2016 for example, household in the second to the poorest wealth quintile position, male household head also have negative indices, whereas household in the richest wealth quintile position, household in the south and urban area have positive concentration indices. Further, the results at the national and regional levels reveal that the majority of observed inequalities in CHE within the three sample periods are attributable to household economic status. Some other positive contributors to CHE inequalities at the national level are sector, and insurance status (see Tables 11, 12, 13).

**Table 12:** Decomposition Analyses of CHE Concentration Index in Urban Region

Variables	Wave 1 (2010/2011)						Wave 2 (2012/2013)						Wave 3 (2015/2016)					
	Coefficient	Mean	Elasticity	Concentration Index (C)	Contribution to C	Contribution to C (%)	Coefficient	Mean	Elasticity	Concentration Index (C)	Contribution to C	Contribution to C (%)	Coefficient	Mean	Elasticity	Concentration Index (C)	Contribution to C	Contribution to C (%)
**Economic Status**																		
Poorest																		
Second	0.0026	0.2169	0.0157	−0.1962	− 0.0030		− 0.0918	0.2247	− 0.0369	− 0.3873	0.0143		− 0.0535	0.2164	− 0.2178	− 0.4312	0.0939	
Middle	− 0.0642	0.1817	−0.3204	0.1495	−0.0479		−0.2176	0.1900	−0.0740	0.0276	−0.0020		−0.0678	0.1932	−0.2464	− 0.0948	0.0233	
Fourth	−0.0709	0.2116	−0.4120	0.2973	−0.1225		−0.1575	0.1939	−0.0546	0.4119	−0.0225		−0.0857	0.2158	−0.3478	0.3372	− 0.1173	
Richest	−0.0794	0.1772	−0.3865	0.8977	−0.3469	95%	−0.4142	0.1972	−0.1462	0.8034	−0.1174	97%	−0.1192	0.1964	−0.4402	0.8846	− 0.3894	99%
**HHsize**																		
< 5 members																		
> 5 members	0.0111	0.3787	0.1156	0.0478	0.0055	−3%	−0.0505	0.5975	−0.0540	−0.0184	0.0009	−1%	0.0211	0.8735	0.3471	−0.0095	− 0.0033	1%
**Gender**																		
Female																		
Male	0.0296	0.4718	0.3837	0.0034	0.0013	−1%	0.0252	0.4600	0.0208	−0.0102	−0.0002	0%	− 0.0221	0.4722	− 0.1967	0.0284	− 0.0056	2%
**Education**																		
No																		
Nursery & primary	0.0028	0.2232	0.0173	−0.0488	−0.0008		0.0164	0.4348	0.0127	0.0322	0.0004		0.0483	0.4493	0.4085	0.0155	0.0063	
Secondary	0.0028	0.0649	0.0051	−0.0034	− 0.0000		− 0.0417	0.3907	− 0.0291	0.0173	−0.0005		0.0993	0.3326	0.6208	−0.0408	−0.0253	
Post-secondary	0.0712	0.0926	0.1814	0.0370	0.0067	0%	−0.1451	0.1659	−0.0431	−0.1318	0.0056	−6%	0.1143	0.1974	0.4240	0.0454	0.0192	9%
**Employment status**																		
Unemployed																		
Employed	−0.0387	0.2279	− 0.2428	− 0.1505	0.0365	−20%	− 0.0960	0.2149	− 0.0369	0.0165	− 0.0006	0%	0.0328	0.2835	0.1748	− 0.0213	− 0.0037	2%
**Bednet**																		
No																		
Yes	−0.0316	0.2614	−0.2269	− 0.0497	0.0112	−6%	− 0.0009	0.2478	− 0.0004	0.0093	−4.0190	0%	0.0076	0.2833	0.0407	− 0.0242	− 0.0009	0
**Hospitalized**																		
No																		
Yes	−0.0507	0.0301	−0.0420	− 0.1589	0.0066	−3%	0.0723	0.0194	0.0025	0.0062	0.0000	− 0%	− 0.0443	0.0194	− 0.0162	− 0.1023	0.0016	− 0%
**Insurance**																		
Uninsured																		
Insured	0.0008	0.1660	0.0038	0.4086	0.0015	−0%	0.2958	0.0534	0.0283	0.3267	0.0092	−10%	− 0.0624	0.0182	− 0.0214	− 0.0417	0.0008	−0%
**Elderly**																		
No																		
Yes	0.0537	0.1496	0.2208	0.0800	0.0176	−9%	−0.0856	0.2034	−0.0311	0.0059	− 0.0001	0%	− 0.0029	0.3907	− 0.0214	0.0367	− 0.0007	0%
CI Ln Odds of CHE					−0.4342						−4.1319						−0.4011	
Mean of Ln Odds of CHE		0.0364						0.5587						0.0532

**Table 13:** Decomposition Analyses of CHE Concentration Index in Rural Region

	Wave 1 (2010/2011)						Wave 2 (2012/2013)						Wave 3 (2015/2016)					
Variables	Coefficient	Mean	Elasticity	Concentration Index (C)	Contribution to C	Contribution to C (%)	Coefficient	Mean	Elasticity	Concentration Index (C)	Contribution to C	Contribution to C (%)	Coefficient	Mean	Elasticity	Concentration Index (C)	Contribution to C	Contribution to C (%)
**Economic Status**																		
Poorest																		
Second	−0.0161	0.1982	−0.1628	− 0.3730	0.0607		− 0.1427	0.1830	− 0.0451	− 0.4169	0.0188		− 0.0796	0.1921	− 0.2863	− 0.3682	0.1054	
Middle	− 0.0472	0.1968	− 0.4715	0.0722	− 0.0340		− 0.3029	0.2066	−0.1081	− 0.0271	0.0029		− 0.0700	0.2010	− 0.2633	0.0551	− 0.0145	
Fourth	−0.0307	0.1984	−0.3092	0.4559	−0.1409		−0.3979	0.2103	−0.1445	0.3899	−0.0563		−0.1131	0.1914	−0.4054	0.5061	−0.2052	
Richest	−0.0425	0.1999	−0.4317	0.7029	−0.3034	90%	−0.5713	0.1999	−0.1973	0.8003	−0.1579	89%	− 0.1184	0.2061	−0.4568	0.6534	−0.2985	92%
**HHsize**																		
< 5 members																		
> 5 members	−0.0071	0.4919	−0.1774	− 0.0227	0.0040	−2%	− 0.0045	0.6626	−0.0052	0.0324	− 0.0001	0%	− 0.0003	0.3939	−0.0055	0.0080	− 0.0000	0%
**Gender**																		
Female																		
Male	−0.0025	0.4999	−0.0652	0.0156	−0.0010	1%	−0.0258	0.4922	−0.0219	− 0.0074	0.0001	− 0%	−0.0046	0.9064	−0.0424	− 0.0249	0.0010	−0%
**Education**																		
No																		
Nursery & primary	−0.0186	0.1816	−0.1718	− 0.0330	0.0056		0.1344	0.4645	0.1078	0.0274	0.0029		−0.0061	0.4427	−0.0510	− 0.0006	0.0000	
Secondary	−0.0206	0.0774	−0.0812	−0.0579	0.0047		0.1849	0.3859	0.1233	−0.0427	−0.0052		−0.0178	0.3766	−0.1255	0.0184	− 0.0023	
Post-secondary	−0.0057	0.1484	−0.0431	−0.0816	0.0035	−5%	0.1226	0.1386	0.0293	0.0250	0.0007	−0%	0.0393	0.1639	0.1208	−0.0075	−0.0009	0%
**Employment status**																		
Unemployed																		
Employed	−0.0023	0.2047	−0.0242	0.0158	−0.0003	2%	−0.0234	0.2015	−0.0081	0.0509	− 0.0004	0%	0.0660	0.1733	0.2143	0.0822	0.0176	−9
**Bednet**																		
No																		
Yes	−0.0153	0.4037	−0.3154	0.0129	−0.0040	2%	0.0552	0.3363	0.0320	−0.0283	−0.0009	1%	0.0133	0.2508	0.0627	−0.0267	−0.0016	0%
**Hospitalized**																		
No																		
Yes	0.0100	0.0224	0.0114	0.1023	0.0011	−1%	0.0602	0.0256	0.0026	0.0235	0.0000	−0%	0.0292	0.0189	0.0103	0.1757	0.0018	−0%
**Insurance**																		
Uninsured																		
Insured	−0.0408	0.0120	−0.0249	0.0999	−0.0024	1%	0.1252	0.0139	0.0030	−0.5044	−0.0015	1	0.0452	0.0058	0.0049	0.0107	0.0000	−0%
**Elderly**																		
No																		
Yes	0.0214	0.1268	0.1383	0.0027	0.0003	−0%	−0.0472	0.1697	−0.0138	−0.0074	0.0001	− 0%	0.0063	0.3284	0.0388	0.0025	0.0000	−0%
CI Ln Odds of CHE					−0.4061						−0.1968						−0.3972	
Mean of Ln Odds of CHE		0.0197						0.0279						0.0534				

## Discussion

Studies in Nigeria decomposing CHE inequality into its contributing socioeconomic factors, together with assessing CHE incidence and intensity, associated factors, inequality concentration and inequality size across several panel NGHS data rounds are scarce. The current study has five crucial findings. First, it observed significant change in the proportion of households facing CHE. Second, it revealed that the main factors associated with CHE are economic status and geopolitical zone. Third, it uncovered that inequality in CHE concentrates among the poor. Fourth, it revealed that inequality size of CHE is reasonably large, but did not significantly change between 2010/2011 and 2015/2016. Fifth, this study disclosed household economic status, sector of residence, and insurance status as the main positive contributors to CHE inequality in Nigeria. These findings are explained in detail below.

First, similar to what was reported in [31], the current study found that the CHE proportion computed using a fixed threshold value is smaller than that calculated utilizing a rank-dependent threshold. At 10% of total household expenditure for instance, the CHE proportion using a fixed threshold value changed from 23 to 43%, whereas the proportion using a rank-dependent threshold value increased higher, from 27 to 48% over the 5-year period. Put differently, the study uncovered that between 2010 and 2016, the share of households experiencing CHE rose from 27 to 48%. This level of CHE proportion is higher than levels reported in some other Sub-Saharan African countries (Ivory Coast, Uganda and Kenya), by [36–38] respectively. This result is, however, expected in a country where a large number of the population, roughly 40.2% live in poverty.

Second, some key factors such as economic status and geopolitical zone were revealed as the major factors associated with CHE in Nigeria. As anticipated, households at the higher economic quintile experiences lesser CHE as opposed to households at lower economic quintile across the three sample periods. This outcome is in line with that of previous studies (e.g., [39; 40]) done in Kenya. Consistent with related earlier work done by [9], the current paper found that households in southern geopolitical zone has higher odds of incurring CHE, as compared to households in northern geopolitical zone of the country in 2015/2016. Large household size was found with less odds of incurring CHE, relative to small household size. As reported in the work of Bhojani et al., [41], the above household size result could be attributed to the fact that households with large number of persons have more income pooling capacity, relative to households with small number of individuals, and as a result experience low CHE levels. This is further strengthened by the work of Adisa, [19], who utilized the probit model to show that households of large size experience reduced CHE. Intuitively, getting more Nigerians to contribute to the insurance scheme will help to make health services affordable to everyone in the country.

Furthermore, households with unemployed household heads experienced increased risk of incurring CHE by roughly 2.2 and 2.7 times in 2012/2013 and 2015/2016, relative to households with employed household heads. This result is not only expected, but in line with already existing studies [40, 42] done in Zambia and Kenya. As in previous studies, employed household heads are financially in a better position, and are able to finance health care cost without experiencing CHE, as compared to unemployed household heads. In terms of health insurance status, the finding of this paper is that insured status of the household is insignificant in determining CHE. This finding is similar to some extant studies [40, 43, 44] in Kenya and Côte d’Ivoire respectively. This further suggests that health care insurance programs in Nigeria have not actually impacted on the risk of CHE (i.e., that it has not reduced CHE), implying the weakness of the programs in financial protection. As in past studies [36, 40], households with elderly members have increased risk of incurring CHE, relative to households without elderly members. While this result was statistically significant in 2010/2011, results of other periods were found insignificant.

Third, the concentration curves revealed that CHE concentrates among the poor across the three periods of time studied. Simply put, there is pro-rich CHE inequality (i.e., inequality that favors’ only the rich) in 2010/2011, 2012/2013 and 2015/2016 in the country. This result is derived from the fact that the concentration curves of 2010/2011 and 2015/2016 dominate the line of equality, as uncovered by the dominance test results in Table 8. Furthermore, the concentration curves of 2010/2011 and 2015/2016 as seen in Fig. 1 above intersect with each other, and it is difficult to judge the extent of inequality and which concentration curve is farther from the line of equality. As reported in the work of [34], this finding implies that none of the two extreme curves dominate each other. In other words, there is no statistically significant dominance between concentration curve of 2010/2011 and that of 2015/2016 in Nigeria. Results of the concentration curves are further supported by those of the concentration indices, reported in Table 10. Here, it is essential to note that the negative concentration indices observed in Table 10 demonstrate that households with low economic status had higher probabilities of incurring CHE, relative to households with high economic status in Nigeria. Interestingly, several past studies [38, 45, 46] have also reported this kind of finding in countries such as Kenya, South Africa and Kenya respectively. Fourth, though the paper revealed reasonably large CHE concentration indices among the poor households as in similar published studies [45, 46], differences between the two extreme sample indices (− 0.2153 in 2010/2011 and − 0.2042 in 2015/2016) where found insignificant. This outcome strengthens that of the concentration curves by simply suggesting that there is no significant change in CHE inequality between 2010/2011 and 2015/2016.

Fifth, the study disclosed household economic status as the main socioeconomic factor that contributes to CHE inequality both nationally and across regions. This suggests that poorer households were more likely to incur CHE, mainly because of their poverty. Other contributors at the national level are sector, and insurance status. The result of household economic status is expected since economic resources are usually unequally distributed between households in the lower and upper part of the income ladder. Past studies [46, 47] in several Sub-Saharan African countries (Kenya, and Democratic Republic of Congo) also support this outcome. Following from this result, policies towards supporting the income of the poor (such as social welfare programmes and subsidies) should be greatly encouraged. This paper also found sector of residence as one of the contributing factors to CHE inequality in Nigeria. Similar to finding(s) in past paper(s) (e.g., [38]), the simple interpretation of the above finding is that inequality in CHE is mainly concentrated among households in the rural part of the country. Hence, government policies to minimize differences in regional welfare distribution could play a great role in saving the rural poor from incurring CHE.

As in the work of Kavosi et al., [34], the current paper found that health insurance status contributed to CHE inequality, though not largely across study periods. This implies that the country’s insurance system has not provided adequate coverage against CHE concentration. This further justifies the above finding that insurance status has not significantly reduced CHE, indicating the weakness of the programme in Nigeria. More so, this low insurance contribution is intuitively similar to what was reported in the works of [40; 44]. For instance, Barasa et al. 2017 reported that health insurance coverage in Kenya remains very low, not adequately covering medical care and hence not protective of catastrophic expenditures. This is similar to findings from a work [44] in Côte d’Ivoire, observing no association between catastrophic expenditures and households having health insurance.

The study has some limitations. First, the OOP data used in this study was collected for a more general medical purpose. For instance, the households provided information on payments made for medicine supply, but it is not clear which particular ill health this medical payment is for. Analyses of OOP for general medical bills conceal information on medical bills for specific ill health, say HIV/AIDS or cardiovascular disease. Second, this study has not analysed forgone health care. Health care is forgone when household is unable to afford needed health services. Household not able to afford health care do not incur CHE, but faces lower quality of life due to untreated health shock. This implies CHE incidence could be underestimated.

Based on these limitations, the current study suggests future areas of research endeavour, as follows. First, future studies could focus on collecting and analysing data on the catastrophic effect of out-of-pocket payments for a specific ill health (e.g., malaria, typhoid fever, HIV/AIDS or cardiovascular disease) in a given state or local government area in the country. Second, future studies could concentrate on analysing the incidence and determinants of forgone health care either for a specific region/area in Nigeria or for the entire country depending on availability of household data.

## Conclusion

This study presents empirical evidences on the extent of dynamics in catastrophic health payments in Nigeria. The study uncovers that catastrophic payments from OOP health expenses in Nigeria remains high, especially among poor households. The catastrophic health payment levels rose from 27% in 2010/2011 to 48% in 2015/2016. Poorer Nigeria households face higher probability of incurring catastrophic health expenditures, relative to richer households. Inequality in catastrophic health expenditures was found to be mainly concentrated among the poor Nigeria households across all studied periods. Following from these findings, concerted effort is needed at reducing the large proportion of OOP present in private health expenditure (PHE) component of total health expenditure (THE), through the enhancement of prepaid health financing mechanisms. This will help in the protection of the poor Nigeria populace from facing financial catastrophe due to health payments. In particular, boosting general revenues to fund public health services adequately is important to ensure effective coverage with quality health care services. Moreover, there is the need to increase coverage of insurance programmes, mainly towards the large number of poor households in the country. These measures will help to place Nigeria on the road to achieving universal health coverage.

## Data Availability

The datasets generated and/or analysed during the current study are publicly available in the World Bank website, through the following link: http://microdata.worldbank.
